# Feasibility and acceptability of a physical activity behavioural modification tele-coaching intervention in lung transplant recipients

**DOI:** 10.1177/14799731221116588

**Published:** 2022-10-28

**Authors:** Emily Hume, Hazel Muse, Kirstie Wallace, Mick Wilkinson, Karen Heslop Marshall, Arun Nair, Stephen Clark, Ioannis Vogiatzis

**Affiliations:** 1 Department of Sport, Exercise and Rehabilitation, Faculty of Health & Life Sciences, 373117 Northumbria University, Newcastle upon Tyne, UK; 2 5983 The Newcastle upon Tyne Hospitals NHS Foundation Trust, Newcastle upon Tyne, UK

**Keywords:** Lung transplantation, tele-rehabilitation, physical activity

## Abstract

**Background:**

Despite improvements in pulmonary function following lung transplantation (LTx), physical activity levels remain significantly lower than the general population. To date, there is little research investigating interventions to improve daily physical activity in LTx recipients. This study assessed the feasibility and acceptability of a novel, 12-weeks physical activity tele-coaching (TC) intervention in LTx recipients.

**Methods:**

Lung transplant recipients within 2 months of hospital discharge were recruited and randomised (1:1) to TC or usual care (UC). TC consists of a pedometer and smartphone app, allowing transmission of activity data to a platform that provides feedback, activity goals, education, and contact with the researcher as required. Recruitment and retention, occurrence of adverse events, intervention acceptability and usage were used to assess feasibility.

**Results:**

Key criteria for progressing to a larger study were met. Of the 15 patients eligible, 14 were recruited and randomised to TC or UC and 12 completed (67% male; mean ± SD age; 58 ± 7 years; COPD *n* = 4, ILD *n* = 6, CF *n* = 1, PH *n* = 1): TC (*n* = 7) and UC (*n* = 5). TC was well accepted by patients, with 86% indicating that they enjoyed taking part. Usage of the pedometer was excellent, with all patients wearing it for over 90% of days and rating the pedometer and telephone contact as the most vital aspects. There were no adverse events related to the intervention. After 12 weeks, only TC displayed improvements in accelerometry steps/day (by 3475 ± 3422; *p* = .036) and movement intensity (by 153 ± 166 VMU; *p* = .019), whereas both TC and UC groups exhibited clinically important changes in physical SF-36 scores (by 11 ± 14 and 7 ± 9 points, respectively).

**Conclusion:**

TC appears to be a feasible, safe, and well-accepted intervention in LTx.

## Introduction

Lung transplantation (LTx) is an established final treatment option for those with end-stage lung disease. Over recent decades, survival rates have improved, with the International Society for Heart and Lung Transplantation Registry reporting a 5-years survival rate of 59%.^1^ In addition to increasing survival, an important goal of LTx is to enhance health-related quality of life (HRQoL) and physical function.^2^ Despite improvements in lung function, significant skeletal muscle weakness and reduced exercise capacity persist after LTx, which may limit improvements in daily physical functioning and HRQoL.^3^ This is due to a host of factors including deconditioning as a result of persistent sedentary time, as well as immunosuppressant medications and episodes of organ rejection which may hinder functional recovery.^4^ Several studies have shown that objectively measured physical activity is significantly reduced in LTx recipients.^5–7^ Collectively, these data are concerning as physical activity is a strong predictor of all-cause mortality, both in patients with chronic respiratory disease and healthy individuals.^8,9^

To date, there is little research investigating interventions to improve daily physical activity in LTx recipients.^2^ One RCT implementing a 12-weeks supervised exercise training programme, demonstrated significantly greater improvements in daily physical parameters compared to usual care.^10^ Although exercise training in the form of pulmonary rehabilitation is recommended for LTx recipients,^11^ access, uptake and completion of these programmes is limited in the UK^12^ and worldwide.^13^ With only six lung transplant centres across the UK, patients often live far away from the transplant centre,^14^ therefore rehabilitation beyond the immediate post-transplant hospital phase is typically only undertaken by a small minority of patients who have a prolonged hospital stay, and this will vary depending on the patient’s geographical location.

Physical activity tele-coaching is a digital intervention that aims to promote physical activity in COPD by facilitating behaviour change techniques such as individually tailored feedback, self-monitoring and goal setting.^15,16^ However, LTx recipients experience significant deconditioning and psychological distress throughout their transplant journey and already have a high treatment burden, involving intensive medication regimes, self-monitoring, diet management and regular hospital appointments^17,18^ Thus, it is not known whether physical activity tele-coaching will be feasible and improve outcomes in these patients. Therefore, the primary objectives of this study were to evaluate: (1) the proportion of LTx recipients accepting participation in the trial; (2) retention of LTx recipients; (3) feasibility of randomisation; (4) participants’ acceptability of the TC intervention and (5) compliance with the intervention and physical activity goals. The secondary objectives were to explore and compare the tele-coaching intervention to usual care to obtain preliminary data on short-term clinical impact and safety of tele-coaching, by measuring physical activity, anxiety/depression and HRQoL outcomes, as well as rates of adverse events.

## Methods

### Ethics approval

This study received ethical approval from the Northeast, Tyne and Wear South Research Ethics Committee (REC Reference 19/NE/0119; IRAS project ID 257479) and was prospectively registered on the clinicaltrials.gov database (NCT03873597).

### Study design

This study was a single centre, parallel two-arm, randomised controlled feasibility study. The trial consisted of three visits, which were all conducted remotely and included: a screening assessment (T0), a baseline assessment (T1) and a post-intervention assessment (12 weeks) (T2).

### Participants

Patients who had undergone single or bilateral LTx and were discharged between February 2020 and October 2021 were recruited from Freeman Hospital, Newcastle upon Tyne NHS Foundation Trust, UK. Potentially eligible patients were identified by designated cardiothoracic transplant co-ordinators, who provided initial information about the trial. Patients received an invitation letter with a participant information sheet and were given time to consider participation in the trial before written informed consent was obtained upon confirmation of eligibility. Patients were consented within 2 months following hospital discharge, to coincide with the first outpatient appointment.

#### Inclusion criteria included

• Undergone single or bilateral LTx with a primary diagnosis of Interstitial Lung Disease (ILD), COPD, Cystic Fibrosis (CF), Bronchiectasis or Pulmonary Vascular Disease.
• Within 2 months of discharge following LTx
• Aged >18 years
• Able to speak and read English.
• Able to provide informed consent.

#### Exclusion criteria included

• Severe post-transplant critical illness neuromyopathy
• Bilateral diaphragmatic weakness
• Presence of any other significant disease or disorder which, in the opinion of the investigators, may either put the participant at risk because of participation in the study, or may influence the result of the study, or the participant’s ability to participate in the study.

### Randomisation and concealment

Participants were assigned to one of two conditions using a computer-generated random sequence, managed by a researcher not involved in the recruitment process. Randomisation (1:1) was stratified by 6MWT distance (6MWD: <300 or ≥300 m),^19,20^ which was performed routinely before hospital discharge, using a block size of two following T1. The tele-coaching group received usual care in addition to the intervention. The control group received usual care, which included a motivational interview session. Given the nature of the intervention, it was not possible to conceal the treatment that participants were assigned to.

### Physical activity tele-coaching intervention

**Figure 1.**
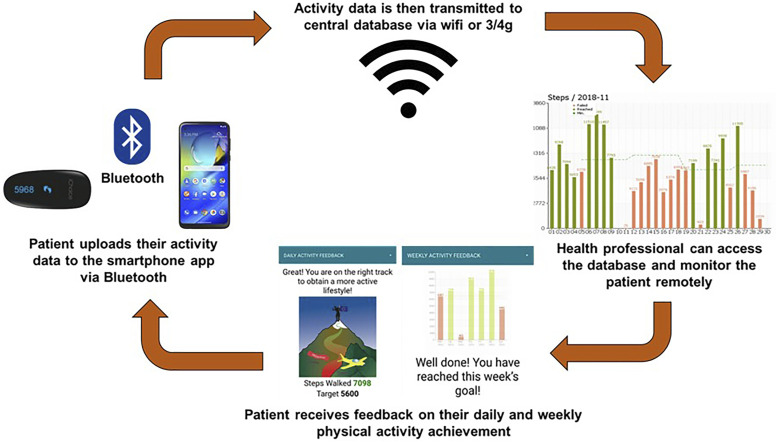
Overview of physical activity behavioural modification tele-coaching intervention.

The 12-week physical activity behavioural modification tele-coaching intervention consisted of a: (1) motivational interview with a coach exploring motivational factors, barriers, preferred and non-preferred activities and strategies to become more active; (2) a pedometer (iChoice Shark A20, Choice MMed America Co., Bristol, PA) providing direct feedback; (3) smartphone app (Linkcare v2.7.1) which uses data collected from the pedometer, transmitted to a smart phone via Bluetooth and simultaneously to the Linkcare web-based platform; (4) home exercise booklet containing general strengthening and stretching exercises in three levels of difficulty and (5) telephone support from the researcher. An overview of the intervention is depicted in Figure 1.

Patients were asked to wear the pedometer during waking hours and interact with the smartphone application every day by reviewing and completing the automated application tasks. Every evening (after 8pm), patients were required to upload their step data to the smartphone application (via Bluetooth) by pressing the button on the pedometer. Each week an activity goal was set by the app, based on the patient’s physical activity levels (steps/day) in the previous week.^16^ The goals were calculated using the mean and median of the 4 most active days.^21^ If the mean value exceeded the weekly goal, the application displayed the option to increase their median goal by 500 steps/day or to keep it the same as the previous week. If the mean value was lower than the weekly goal and the median was more than 500 steps/day below the goal, the goal was reduced to the median of the 4 most active days +500 steps/day.^16^ Otherwise, the goal remained the same. The app also provided patients with daily feedback, encouragement, and educational messages, which were displayed in text or picture format. Throughout the intervention, researchers could access patient data via their app linked web-based platform (Linkcare app v2.7.1, Caldicott approval: 7372) and monitor their physical activity progress and adherence to the intervention. Telephone contact from the researcher was triggered if patients: (1) did not send their step count data for three consecutive days, (2) did not reach their step target for 2 consecutive weeks, (3) reached the step target but were not willing to increase their goal for 2 consecutive weeks. Prior to commencing the intervention, all patients received an instruction guide on how to use the smartphone application.

### Usual care

Usual care for LTx recipients included physical mobilisation whilst in the intensive care unit and post-transplant ward. During this time, patients were provided with a set of individualised rehabilitation exercises to conduct at home following hospital discharge. Additionally, as part of the study, participants assigned to usual care underwent a motivational interview to encourage patients to be physically active. This included education on the benefits of being physically active, goal setting and self-monitoring of physical activities.

### Outcomes to assess feasibility

A priori progression criteria were used to consider whether it would be appropriate to progress to a full-scale study. Based on other similar feasibility studies^22-24^ these included: (1) feasibility to recruit participants, (2) retention of participants, (3) feasibility of randomisation processes, 4) intervention acceptability, and (5) intervention usage (Online supplement, Table 1).

**Table 1.** Characteristics of patients at baseline (hospital discharge).

Characteristic	Tele-coaching (*n* = 7)	Usual care (*n* = 5)
Age (years)	57 ± 9	58 ± 4
BMI (kg/m^2^)	22.7 ± 3.9	25.3 ± 2.9
Sex (male/Female)	4/3	4/1
FEV_1_ (L)	2.22 ± 0.52	2.15 ± 0.67
FEV_1_ (% predicted)	70 ± 11	73 ± 21
FVC (% predicted)	66 ± 11	73 ± 27
FVC (L)	2.56 ± 0.74	2.75 ± 1.12
FEV_1_/FVC %	88 ± 7	81 ± 10
6MWD (m)	325 ± 69	336 ± 43
Diagnosis		
COPD	3	1
CF	1	0
ILD	2	4
PAH	1	0
Hospital length of stay (days)	45 ± 23	38 ± 4

BMI = Body mass index, COPD = Chronic Obstructive Pulmonary Disease, CF = Cystic Fibrosis, ILD = Interstitial Lung Disease, PAH = Pulmonary Arterial Hypertension. Values are mean ± SD.

### Criterion 1: Screening, eligibility and recruitment

The screening rate was defined as the number of patients that were approached by the research team and assessed for eligibility against the inclusion and exclusion criteria. This included those who decided not to take part. Eligibility was determined by dividing the number of people screened by the number who met the inclusion criteria.

The research team recorded all patients that met the eligibility criteria and decided not to take part in the trial, along with the reason for their decision.

### Criterion 2: Retention

The retention rate was defined as the number of participants who remained in the study and did not drop out.

### Criterion 3: Randomisation feasibility

Randomisation feasibility was assessed by the number of participants that were willing to be randomised to either the intervention or usual care group.

### Criterion 4: Patient acceptability

Acceptability of the intervention by patients was assessed through a project specific questionnaire at T2,^15^ consisting of 16 multiple choice questions on their experiences with the intervention, including 10-point Likert scales to rate the usefulness of the intervention components. Patients were asked to complete this 15-min questionnaire at T2.

### Criterion 5: Actual usage of the intervention and step goal compliance

Actual usage of the pedometer throughout the intervention was assessed objectively using the data on the web based LinkCare Platform, specifically the pedometer readings on a day-to-day basis. Usage of the pedometer was determined by the presence of step count data (>70 steps for that day),^15,16^ to verify actual usage of the pedometer each day. Compliance with the step goal was assessed using the step data and goals set on the platform. Self-reported usage of the pedometer and home exercise booklet was also assessed within the acceptability questionnaire.

### Contact time

All contact with patients was recorded in a case file, including details on the duration and reason for each contact.

### Adverse events

An adverse event was defined as any untoward occurrence that occurred during the conduct of the study. All adverse events were recorded in the adverse event log within the patients notes and were classified as serious or not, and attributable to the study or not, as per the ‘Decision Tree for Adverse Event reporting’ from the National Institute for Health Research, Clinical Research Network, Introduction to Good Clinical Practice Toolkit^25^

### Outcomes to assess clinical effectiveness

#### Physical activity

Physical activity was assessed objectively using an Actigraph accelerometer (Actigraph LLC Pensacola, Florida, USA) in the week following T1 and the week following T2. This accelerometer has been previously validated in patients with COPD.^26^ Patients in both the tele-coaching and usual care groups were instructed to wear the accelerometer for seven consecutive days during waking hours. The accelerometer was positioned using an elasticated waistband on the participant’s dominant side on the iliac crest at the anterior axillary line. Prior to wearing the accelerometer, participants were given written instructions with a visual demonstration on: (1) the correct positioning of the device; (2) the start and end date of the physical activity assessment; (3) the wearing period (I.e. wear the device during waking hours); (4) when the device should be removed (I.e. during water based activities such as showering or bathing). A valid physical activity measurement was defined as a minimum of four weekdays, with at least 8 hours of wear time. Weekends were excluded from the analysis, in line with taskforce recommendations for COPD patients.^27^ The physical activity parameters assessed included daily steps, movement intensity, time spent in sedentary and at least light activity intensities.

The pedometer was used by the intervention group as part of the tele-coaching intervention, to provide direct feedback to patients on their daily steps.

### Additional assessments

Additional outcomes assessed at T1 and T2 included: (1) HRQoL through the SF-36 questionnaire and (2) Anxiety and Depression using the Hospital Anxiety and Depression Scale (HADS).^10^

### Analyses

All statistical analyses were performed using SPSS version 27 (IBM, UK). Prior to analysis, the assumption of normality for outcomes was assessed using the Shapiro Wilk Test. Descriptive statistics were reported to better understand the distribution and potential for change of the proposed outcomes.

Data from the project-tailored questionnaire were scored as categorical variables and reported as frequencies and percentages (number of patients indicating each answer), except for the usefulness ratings of the components, which were expressed as medians (IQR). Actual usage of the pedometer was expressed as the percentage of patients who wore the pedometer for at least 90% of the days, as well as the median (IQR) wear time (days per week). The 90% cut off point was derived from a study utilising a similar intervention in COPD patients,^15^ to allow comparison between studies. Weekly compliance to the goal was presented as the percentage of goals met over the intervention period (12 weeks).

The feasibility study was not powered to test the effectiveness hypotheses associated with any planned main large-scale trial. Paired t-tests or Wilcoxon Signed Ranks Test were employed to assess the within group differences from T1 to T2, to identify whether the intervention or natural recovery had a significant effect on physical activity outcomes. Independent samples t-tests or Mann-Whitney U tests were used to detect differences in change scores (∆) between groups. Statistical significance was set at *p* < .05 for all analyses.

## Results

### Participants

**Figure 2.**
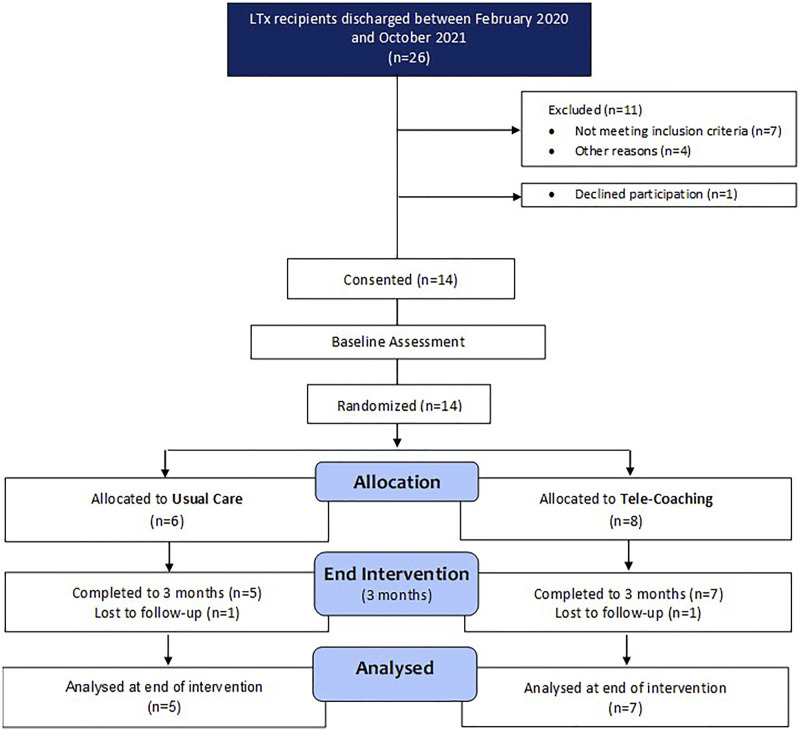
CONSORT Participant Flow Diagram.

In total, 14 LTx recipients provided consent for the study and were randomised to the tele-coaching intervention (*n* = 8) or usual care (*n* = 6). Twelve patients completed T2 (Figure 2, Table 1).

### Criterion 1: Screening, eligibility and recruitment

A total of 26 LTx recipients were discharged between February 2020 and October 2021. Of those 26, four were unable to be approached, due to the suspension of trial recruitment at the start of the COVID-19 pandemic. In total, 22 patients were screened by accessing patient records or by direct contact in clinic. Of the 22 patients screened, 7 (32%) were not eligible to participate in the trial. The remaining 15 patients received information about the trial (Figure 2).

In total, 14 LTx recipients were recruited between February 2020 and October 2021. No patients were recruited from March to October 2020, as well as mid-January to May 2021 due to the suspension of LTx in response to the COVID-19 pandemic.^28^ The consent rate for the study was high at 93%, with 14 out of 15 patients accepting participation.

### Criterion 2: Retention

The retention rate was 86% for patients that consented to take part in the study. The dropout rates were equal between the tele-coaching and usual care group and the reasons for drop out were: (1) extenuating personal circumstances and (2) chronic lung allograft dysfunction resulting in palliative care.

### Criterion 3: Randomisation

All 14 patients were willing to be randomised to either the intervention or usual care group following T1.

### Criterion 4: Acceptability of intervention

**Figure 3.**
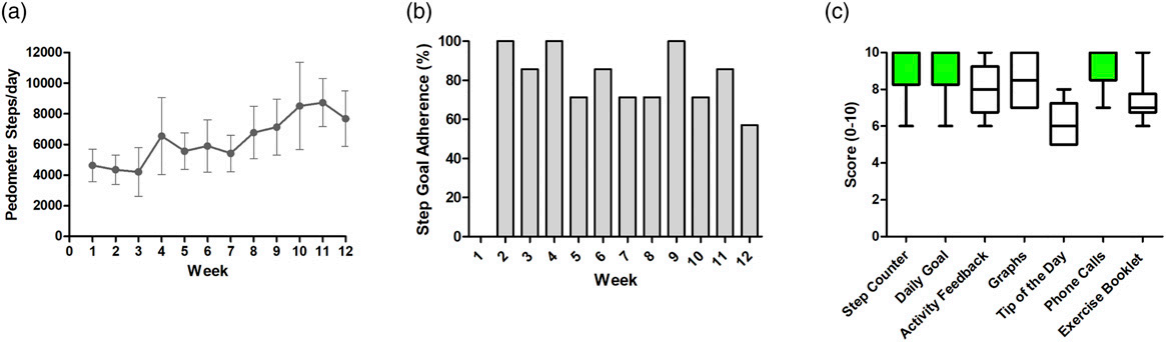
(A) pedometer steps/day, (B) step goal compliance and (C) boxplots depicting the usefulness scores (1-10 likert scale) of the different intervention components rated by patients.

Overall, patient feedback on the intervention was positive, with 86% of patients indicating that they either “liked” (29%) or “liked the intervention a lot” (57%) (Table 2, Figure 3). Furthermore, 86% of patients reported that the intervention “helped them a lot” to improve their physical activity levels, with 86% of patients indicating that the smartphone app was either “very easy” or “easy” to use. Importantly, 86% of patients were willing to use at least one aspect of the intervention in the future.

**Table 2.** Overview of patient responses from acceptability questionnaire.

Question 1)	Liked it a lot	Liked it	Neutral	I disliked it	No opinion
How much did you enjoy taking part in the activity programme?	57%	29%	14%	0%	0%

Question 2)	Yes, helped a lot	Yes, helped a little	Not noticeable	No, not at all	No, it discouraged me
Did the intervention help you to increase your physical activity levels?	86%	0%	14%	0%	0%

Question 3)	Much too low	A little bit too low	Reasonable	A little bit too high	Much too high
How did you experience the weekly goal increases during the intervention?	0%	0%	86%	14%	0%

Question 4)	Very easy	Easy	Not easy, but managed	Difficult	Very difficult
How was it for you to work with the smartphone intervention?	29%	57%	0%	14%	0%

Question 5)	Step counter	Smartphone app	Telephone contact	Exercise booklet	Other
In your opinion, what was the most important part of the intervention?	57%	0%	43%	0%	0%

Question 6)How often did you perform the following actions?	Several times per day	Once per day	Sometimes but not everyday	Once or twice per week	Never
a) Look at the step counter	86%	14%	0%	0%	0%
b) Do any home exercises	57%	14%	0%	0%	29%

Question 7)	Very helpful and supportive	Helpful and supportive	Neutral	Poor, not supportive	Very poor, not supportive at all
How would you rate the graphics used on the smartphone application?	0%	57%	29%	14%	0%

Question 8)	Very quick	Quick	Neutral	Slow	Very slow
How would you rate the interaction between you and the app?	0%	43%	43%	0%	14%

Question 9)	Nothing	Step counter	Step counter, phone and feedback messages	Step counter, phone and contact	Whole intervention
Which part of the intervention would you be willing to use in the future?	14%	14%	0%	29%	43%

### Criterion 5: Actual usage of the intervention and step goal compliance

Of those completing the intervention, 100% wore the pedometer for more than 90% of days over the 12-weeks intervention. Overall, patients wore the pedometer for a median of 7 (IQR: 7–7) days per week.

In terms of self-reported usage, 86% of patients indicated that they looked at the pedometer “several times a day,” the remaining 14% indicated “once daily.”

The number of weekly step goal targets met throughout the 12-weeks intervention was good, with a mean (SD) of 82 ± 14% of step goals achieved (Figure 3).

### Contact time

The total mean ± SD contact time per patient was 52 ± 23 min per patient. On average, patients had to be contacted 9 ± 4 times over the 12-weeks. If the patient was progressing well and no contact was triggered, general well-being checks were conducted every 2 weeks via brief phone calls. For instances where the patient did not send their step data for three consecutive days, did not reach their step target for 2 consecutive weeks, or chose not to increase their goal for 2 consecutive weeks, the mean number of contacts was increased as well as the time for consultation. This was to provide troubleshooting solutions and explore barriers of engagement with goal adjustment.

### Adverse events

Over the study period, there were no adverse events related to the intervention and the effort of patients to progressively increase their activity levels, or related to the study protocol or procedures.

### Hospital admissions and complications

Throughout the 12-week intervention period, six patients (Tele-Coaching: *n* = 4 and Usual Care: *n* = 2) were admitted to the hospital for more than 72 h. In the tele-coaching group, the reasons for admission were acute rejection resulting in reduction in pulmonary function (*n* = 3), fever and suspected infection (*n* = 1) and dyspnoea due to right main bronchus anastomotic stricture (*n* = 1). In the usual care group, hospital admissions were for acute rejection resulting in reduction in pulmonary function (*n* = 1) and acute kidney injury (*n* = 1).

### Outcome measures

#### Accelerometer-derived physical activity

At 12 weeks there were clinically important^29^ improvements in steps/day for both the tele-coaching (by 3475 ± 3422 steps/day; *p* = .036) and usual care (by 1159 ± 991 steps/day; *p* = .059) groups, however the increase in the tele-coaching group exceeded the usual care group by clinically important margins^29^ (by 2316 steps/day) (Table 3).

**Table 3.** Changes in PA parameters and HRQoL outcomes in the Tele-Coaching and Usual Care groups.

Outcome	Group	Baseline (T1)	12 weeks (T2)	Within group change	Between group p value
Accelerometer Outcomes					
Daily steps (steps/day)	TC	3558 ± 3188	7033 ± 5944	3475 ± 3422^*#^	0.089^#^
	UC	4249 ± 3531	5408 ± 4444	1159 ± 991^#^	
Movement intensity (VMU)	TC	237 ± 155	390 ± 311	153 ± 166^*^	0.037
	UC	317 ± 153	314 ± 129	−3 ± 61	
Time spent in sedentary activity (min/day)	TC	570 ± 108	513 ± 296	−57 ± 128	0.114
	UC	441 ± 59	463 ± 85	22 ± 52	
Time spent in at least light activity (min/day)	TC	160 ± 58	197 ± 72	37 ± 24^*^	0.040
	UC	191 ± 71	194 ± 58	3 ± 37	
HADS					
Anxiety	TC	5 ± 4	4 ± 4	1 ± 4	0.339
	UC	3 ± 4	4 ± 5	1 ± 2	
Depression	TC	3 ± 4	4 ± 4	0 ± 5	0.479
	UC	3 ± 3	3 ± 5	0 ± 2	
SF-36					
PCS Score	TC	28 ± 11	39 ± 14	11 ± 14^#^	0.291
	UC	30 ± 7	37 ± 12	7 ± 9^#^	
MCS score	TC	57 ± 7	53 ± 14	−4 ± 15	0.348
	UC	51 ± 14	51 ± 13	−1 ± 10	

Abbreviations: VMU = Vector Magnitude Units, MVPA = moderate to vigorous physical activity, HADS = Hospital Anxiety and Depression Scale, SF-36 = Short Form 36 Questionnaire, PCS = Physical Component Summary, MCS = Mental Component Summary, TC = Tele-Coaching, UC = Usual Care.

Values are mean ± SD ^*^ = statistically significant (P<0.05), ^#^ = clinically important change.

**Figure 4.**
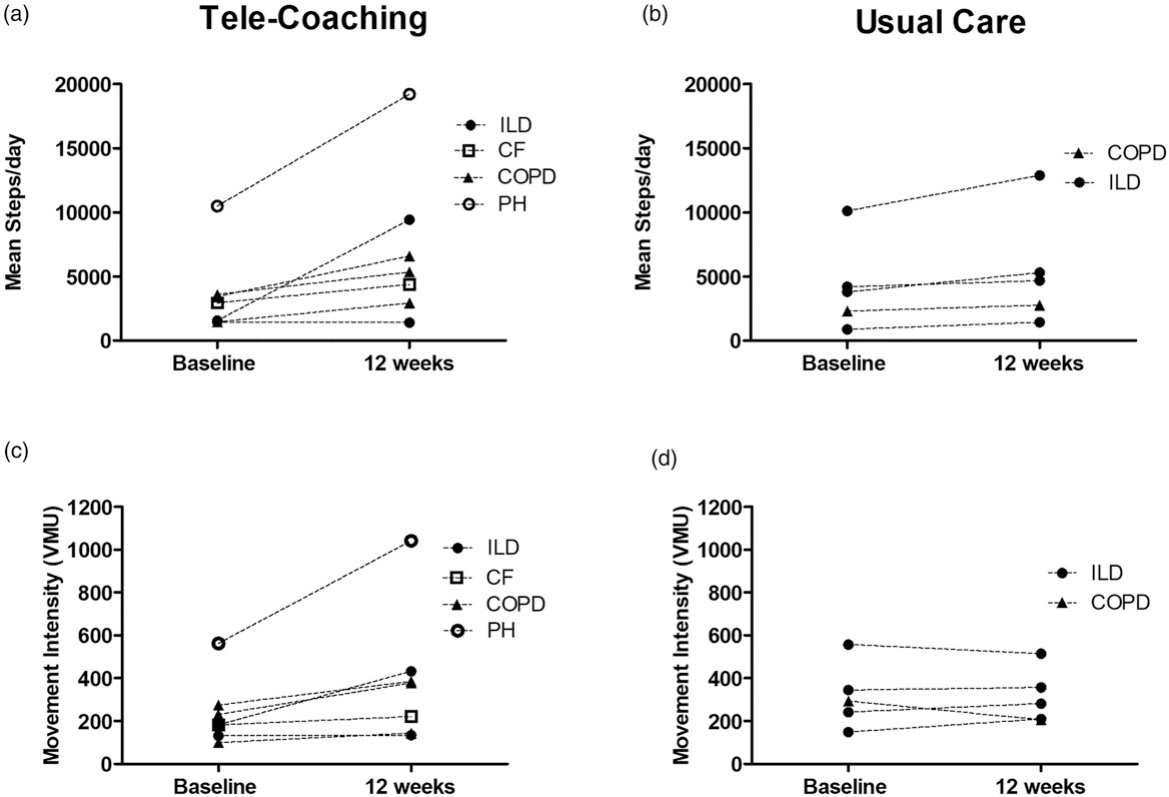
Individual changes in steps/day and movement intensity (VMU) in the tele-coaching (A&C) and usual care (B&D) groups from baseline to 12 weeks.

Accelerometer movement intensity significantly improved within the tele-coaching group (by 153 ± 166 VMU; *p* = .019), but not the usual care group (by −3 ± 61 VMU; *p* = .908), with a significant difference between groups (by 156 VMU; *p* = .037). For time spent in at least light activity, there was a significant increase within the tele-coaching group (by 37 ± 24 min/day; *p* = .006) at 12 weeks, but not in the usual care group (by 3 ± 37 min/day; *p* = .861), with a significant difference between groups (by 34 min/day; *p* = .040). Individual changes in steps/day and movement intensity for each disease entity in the tele-coaching and usual care groups are presented in Figure 4. For daily steps and movement intensity the mean improvement in the tele-coaching group was 3896 ± 5580 steps/day and 127 ± 175 VMU, respectively for ILD (*n* = 2), 2126 ± 919 steps/day and 100 ± 52 VMU, respectively for COPD (*n* = 3), 8717 ± 0 steps/day and 479 ± 0 VMU, respectively for Pulmonary Arterial Hypertension (PAH) (*n* = 1) and 1438 ± 0 steps/day and 39 ± 0 VMU, respectively for CF (*n* = 1). For usual care, the mean improvement in daily steps and movement intensity were 1329 ± 1057 steps/day and 17.8 ± 44.7 VMU, respectively for ILD (*n* = 4) and 479 ± 0 steps/day and −87.9 ± 0 VMU, respectively for COPD (*n* = 1).

#### HRQoL and psychological wellbeing

At 12 weeks, there were clinically important (>2–3 units^30^) increases in SF-36 physical component summary scores, in both the tele-coaching (+11 points) and usual care (+7 points) groups, however these changes were not statistically significant between groups.

## Discussion

This study showed that tele-coaching was feasible, safe, and well accepted by LTx recipients. Patient uptake and retention, acceptability and usage of the tele-coaching intervention was high, without occurrence of adverse events. When compared to usual care, tele-coaching elicited improvements in accelerometer derived physical activity parameters that exceeded clinically important margins, highlighting the potential efficacy of this intervention to support patients post LTx.

Recruitment for the trial was significantly affected by the COVID-19 pandemic and the suspension of LTx during the early stages of the pandemic. Thus, the main reason for slow patient recruitment was due to the limited number of transplants performed. A centre-specific investigation reported this as a 77% reduction during the first peak of the pandemic.^28^ Although the number of transplants was limited, uptake of the study was high with 93% of eligible participants accepting participation. This well exceeds criteria previously used to proceed to a full-scale trial (>30% of eligible patients recruited).^22^ Additionally, there were low rates of attrition in both the tele-coaching and usual care groups (14% overall) over the 12-weeks. According to previous literature, attrition of <20% is unlikely to threaten the validity of a trial.^31^ Additionally, this is significantly lower than the dropout rate previously reported in a meta-analysis of app-based interventions in chronic disease.^32^

Overall, the tele-coaching intervention was well accepted by patients, who rated their enjoyment similarly to a study using the same intervention in COPD patients.^15^ Most patients (86%) reported that the intervention ‘helped them a lot’ to improve their physical activity, which is higher than that previously reported in COPD patients (59%).^15^ The simplicity of the smartphone application may have contributed to the good acceptability of the intervention, as most patients reported finding it easy to use. In COPD patients, 47.8% rated the goal increases as either ‘high’ or ‘much too high’ compared to only 14% in the current study in LTx recipients, which is supported by high step goal compliance (82 ± 14%).^15^ This may suggest that LTx recipients are more ambitious with their physical activity targets, because of improved lung function and diminished symptoms of breathlessness.^33^

Alike to the findings in COPD patients,^15^ LTx recipients considered the pedometer and telephone contact with the researcher as the most important components of the intervention. The regular contact with the researcher to resolve and advise on any safety concerns in the current study, may have enhanced patient’s self-efficacy to undertake more physical activity,^34,35^ and highlights the significance of a collaborative approach between the patient and healthcare professional (HCP) in facilitating patient behaviour change and self-management.^36^

Although HCP contact was important, the average contact time required for each patient was only 52 min over the 12-weeks intervention. In the current study, coaching eight patients simultaneously over 12 weeks, would equate to around 35 min of HCP time per week, which is significantly less resource intensive than pulmonary rehabilitation. The low contact time could have been facilitated by several factors, such as the semi-automated nature of the intervention, the instruction booklet provided to help with working the app, as well as the simplicity of the app, as 86% of patients indicated that they found the app either “very easy” or “easy” to use.

The high-level of perceived importance of the pedometer by patients was also reflected by the excellent actual and self-reported usage of the pedometer. All patients wore the pedometer for over 90% of the 12-weeks programme, which was higher than that previously reported in the study in COPD patients (59%).^15^

In terms of accelerometer physical activity outcomes, there were clinically important improvements in steps/day in both groups. The improvement in the usual care group highlights the natural recovery occurring in the early stages of LTx recovery, similarly to Langer et al.^10^ who demonstrated an improvement of 750 steps/day in a usual care group within an exercise training study. A recent systematic review^37^ highlighted that the majority of rehabilitation studies conducted post-transplantation are limited by the lack of a control group, making it difficult to differentiate the true effect of the intervention. Literature on interventions to improve physical activity in LTx recipients is scarce.^2^ Improvements in daily steps in the current study exceeded those shown following exercise training.^10,38^ This is likely due to step counts being the central focus of the intervention and the incorporation of behavioural techniques such as self-monitoring, goal setting and feedback, which have been deemed important for enhancing healthy activity behaviours.^39^

Although peripheral muscle abnormalities have been shown to be the predominant limiting factor to exercise capacity in lung transplant recipients,^3^ the underlying lung disease entity and pathophysiology may also influence an individual’s exercise capacity and physical activity behaviour. When examining individual changes following tele-coaching (Figure 4), the largest improvements in daily steps and movement intensity were seen in PAH, whereas the lowest was in CF. Although CF recipients are often younger compared to other disease entities such as COPD, CF is a multi-organ disease in which co-morbities such as diabetes mellitus and bone disease are common both pre- and post-transplant.^40^ It is important to note that no conclusions can be drawn from this data due to the limited sample size, however this poses an interesting question for future research. Our findings on HRQoL support those of Finlen Copeland et al.,^41^ showing that SF-36 PCS scores demonstrate a natural course of recovery following LTx, likely due to improved pulmonary function, symptoms and ability to perform daily activities. The lack of change in HADs and SF-36 MCS scores in either group reflects previous findings.^10,42^ This may be due to several factors such as the uncertainty of organ rejection, adverse effects of immunosuppressive medications and recurring pain following LTx. However, a full scale RCT is required to infer whether tele-coaching can induce greater improvements in HRQoL outcomes than usual care alone.

### Implications of the study

This study highlights the potential of digital health technology to increase physical activity levels in LTx recipients in the early stages of recovery. The present study may inform a fully powered RCT to determine whether a digital physical activity intervention can elicit significantly greater improvements in physical activity and HRQoL outcomes compared to usual care, as well as determine the longer-term impact of this intervention.

### Study limitations

There are several limitations that must be considered in this study. Firstly, this was a small-scale study, therefore, generalisability of the results to clinical practice may be limited. However, the main aim of this study was to explore the feasibility and acceptability of tele-coaching in LTx recipients, thus it was not powered to detect differences in study outcomes between groups. Secondly, acceptability of the intervention was assessed through a project specific questionnaire which was used previously by Loeckx et al.^15^ in COPD patients. This makes it challenging to make comparisons with other studies implementing digital health interventions, however it provides useful insights into patient acceptability and can be compared to the findings by Loeckx et al.^15^ to explore differences between different patient groups using the same intervention. Finally, randomisation to the tele-coaching and usual care groups was stratified based on functional exercise capacity (6MWD) as this has been demonstrated as a strong predictor of physical activity change,^43^ consequently it was not possible to balance groups for all variables (e.g. sex and disease entities) and there was a large diversity of primary disease diagnosis and therefore underlying pathophysiology of physical activity limitation.^44^

## Conclusion

In conclusion, physical activity tele-coaching appears to be a feasible, safe, and acceptable intervention to support patients post LTx. Additionally, there is a degree of natural recovery in some physical activity and HRQoL parameters, however tele-coaching appears to elicit greater improvements in physical activity measures.

## Supplemental Material

Supplemental Material - Feasibility and acceptability of a physical activity behavioural modification tele-coaching intervention in lung transplant recipients Click here for additional data file. Supplemental Material for Feasibility and acceptability of a physical activity behavioural modification tele-coaching intervention in lung transplant recipients by Emily Hume, Hazel Muse, Kirstie Wallace, Mick Wilkinson, Karen Heslop Marshall, Arun Nair, Stephen Clark, and Ioannis Vogiatzis in Chronic Respiratory Disease
