# Supplementary material for: Feasibility and acceptability of a physical activity behavioural modification tele-coaching intervention in lung transplant recipients
Source: Chron Respir Dis. 2022 Oct 28;19:14799731221116588. doi: 10.1177/14799731221116588 (PMC9619269; doi:10.1177/14799731221116588)
Supplement: Supplemental Material - Feasibility and acceptability of a physical activity behavioural modification tele-coaching intervention in lung transplant recipients [file sj-pdf-1-crd-10.1177_14799731221116588.pdf]

ONLINE SUPPLEMENT

Results:

Table 1: Summary of progression criteria for feasibility outcomes

**Progression Criteria**	**Assessment of Criteria**	**Outcome**	**Decision**
1) Feasibility to recruit a sufficient proportion of LTx recipients.	Recruitment: percentage of eligible patients recruited; if > 30% recruited = proceed, if < 10% = unlikely to be feasible; if 10–30% = CI to consider feasibility of proceeding based on screening rate and possible steps to increase recruitment.[1, 2]	Recruitment: 15 were eligible (22 were screened); 93% of eligible patients (63% of those screened) were recruited.	Proceed
2) Retention to 12-week follow-up (T2).	Retention: percentage of participants retained; if > 80% = proceed, if < 60% = unlikely to be feasible, if 60–80% = CI to consider feasibility of proceeding based on available data and possible steps to increase retention.[1, 3]	Retention: 86% of participants enrolled in the study were retained.	Proceed
3) Randomisation Feasibility	>80% of participants randomised to the intervention or usual care following baseline assessment. [1]	All patients consented (100%) were randomised to either to tele-coaching or usual care group following their baseline assessment.	Proceed
4) Acceptability of intervention	Intervention acceptability was considered by a project specific questionnaire and compared to previous findings in COPD patients. [4]	Acceptability of the intervention was good (see Table 2). 86% enjoyed taking part in the programme, 86% willing to use at least one aspect of the intervention in the future.	Proceed
5) Intervention Usage	Actual usage of pedometer was defined as presence of >70 steps for that day present on the LinkCare Platform. [4]	All patients wore the pedometer for >90% of days.	Proceed

Online Supplement References

1. Ward N, Stiller K, Rowe H, et al. Airway clearance by exercising in mild cystic fibrosis (ACE-CF): A feasibility study. *Respir Med* 2018; 142: 23-28. 2018/09/02. DOI: 10.1016/j.rmed.2018.07.008.
2. Haines M. Feasibility of procedures for a randomised pilot study of reduced exertion, high-intensity interval training (REHIT) with non-diabetic hyperglycaemia patients. *Pilot and Feasibility Studies* 2020; 6: 28. DOI: 10.1186/s40814-020-00571-8.
3. Hawkins J, Charles JM, Edwards M, et al. Acceptability and Feasibility of Implementing Accelorometry-Based Activity Monitors and a Linked Web Portal in an Exercise Referral Scheme: Feasibility Randomized Controlled Trial. *J Med Internet Res* 2019; 21: e12374. 2019/03/30. DOI: 10.2196/12374.
4. Loeckx M, Rabinovich RA, Demeyer H, et al. Smartphone-Based Physical Activity Telecoaching in Chronic Obstructive Pulmonary Disease: Mixed-Methods Study on Patient Experiences and Lessons for Implementation. *JMIR Mhealth Uhealth* 2018; 6: e200. 2018/12/24. DOI: 10.2196/mhealth.9774.
